# The triglyceride-glucose index as a potential protective factor for hypertrophic obstructive cardiomyopathy without diabetes: evidence from a two-center study

**DOI:** 10.1186/s13098-023-01084-z

**Published:** 2023-06-29

**Authors:** Xiangbin Meng, Jun Gao, Kuo Zhang, Wen Jun, Jing-Jia Wang, Xu-Liang Wang, Yuan-Geng-Shuo Wang, Ji-Lin Zheng, Yu-Peng Liu, Jing-Jing Song, Jie Yang, Yi-Tian Zheng, Chen Li, Wen-Yao Wang, Chunli Shao, Yi-Da Tang

**Affiliations:** 1 Department of Cardiology and Institute of Vascular Medicine, Key Laboratory of Molecular Cardiovascular Science, Ministry of Education, Peking University Third Hospital, No.49 Huayuanbei Road, Beijing, 100191 China; 2 Department of Cardiology, State Key Laboratory of Cardiovascular Disease, Fuwai Hospital, National Center for Cardiovascular Diseases, Chinese Academy of Medical Sciences and Peking Union Medical College, Beijing, 100037 China; 3 Department of Cardiology, Guangdong Provincial People’s Hospital (Guangdong Academy of Medical Sciences), Southern Medical University, Guangzhou, China

**Keywords:** HOCM (Hypertrophic obstructive cardiomyopathy), TyG (Triglyceride-glucose Index), Glucose metabolism, Prognosis

## Abstract

**Objective:**

This study aimed to investigate the relationship between the TyG (Triglyceride-glucose index) and the prognosis of patients with HOCM (hypertrophic obstructive cardiomyopathy) without diabetes.

**Research design and methods:**

A total of 713 eligible patients with HOCM were enrolled in this study and divided into two groups based on treatment: an invasive treatment group (n = 461) and a non-invasive treatment group (n = 252). The patients in both two groups were then divided into three groups based on their TyG index levels. The primary endpoints of this study were Cardiogenic death during long-term follow-up. Kaplan–Meier analysis was used to study the cumulative survival of different groups. Restricted cubic spline was used to model nonlinear relationships between the TyG index and primary endpoints. Myocardial perfusion imaging/Myocardial metabolic imaging examinations were performed to assess glucose metabolism in the ventricular septum of the HOCM patients.

**Results:**

The follow-up time of this study was 41.47 ± 17.63 months. The results showed that patients with higher TyG index levels had better clinical outcomes (HR, 0.215; 95% CI 0.051,0.902; P = 0.036, invasive treatment group; HR, 0.179; 95% CI 0.063,0.508; P = 0.001, non-invasive treatment group). Further analysis showed that glucose metabolism in the ventricular septum was enhanced in HOCM patients.

**Conclusions:**

The findings of this study suggest that the TyG index may serve as a potential protective factor for patients with HOCM without diabetes. The enhanced glucose metabolism in the ventricular septum of HOCM patients may provide a potential explanation for the relationship between the TyG index and HOCM prognosis.

## Highlights

- The study aimed to investigate the relationship between the TyG index and prognosis in HOCM patients without diabetes.
- The TyG index was found to be a potential protective factor in HOCM patients without diabetes, with higher TyG index levels associated with better clinical outcomes.
- Enhanced glucose metabolism in the ventricular septum may explain the relationship between the TyG index and HOCM prognosis.
- The findings of this study may provide useful information for the clinical management of HOCM patients.

## Background

Hypertrophic cardiomyopathy (HCM) is a genetic heart disease characterized by hypertrophy of the left ventricular myocardium, and it is a common cause of sudden cardiac death in young adults [1]. The incidence of HCM is approximately 1 in 500 individuals. The current treatment for HCM is mainly symptomatic and supportive, and the long-term prognosis is affected by various factors such as age, the presence of comorbidities, and the severity of the hypertrophy [2–8]. Therefore, exploring new prognostic factors and potential therapeutic targets is of great significance for improving the prognosis of HCM.

Under the condition of increased left ventricular pressure load, myocardial metabolism may undergo significant changes. The energy substrate of a healthy heart is mainly fatty acid. When the heart is under high load, ATP produced by fatty acid oxidation cannot adapt to the increase of heart energy consumption, and glucose oxidation with higher productivity will prevail. This energy substrate transformation is an adaptive metabolic remodeling, which helps to protect damaged myocardium, avoid further damage, and supply energy with higher efficiency [9], and this metabolic remodeling may have a protective effect on the damaged myocardium [10]. In hypertrophic obstructive cardiomyopathy (HOCM), the significant increase in left ventricular outflow tract pressure difference and the corresponding alterations in hemodynamics may be more pronounced in terms of alterations in myocardial energy metabolism [9, 11, 12].

The Triglyceride Glucose (TyG) Index, a novel indicator of insulin resistance, has been shown to be closely related to cardiovascular disease [13–16]. The TyG Index is a simple and reliable marker of insulin resistance that can be calculated based on the levels of triglycerides and glucose in the blood. Elevated TyG Index has been shown to be associated with an increased risk of cardiovascular disease, including myocardial infarction, heart failure, and stroke. However, to date, no studies have explored the role of the TyG Index in HCM.

In this study, we aimed to investigate the potential role of the TyG Index in predicting the prognosis of HOCM without diabetes. We also performed a radionuclide examination to investigate the metabolic changes in the ventricular septum of HCM patients, and further exploration that enhanced glucose metabolism may play a role in the effect of the TyG Index on HOCM prognosis.

## Research design and methods

### Design, patients, and outcome measure

#### Study patients

**Fig. 1:**
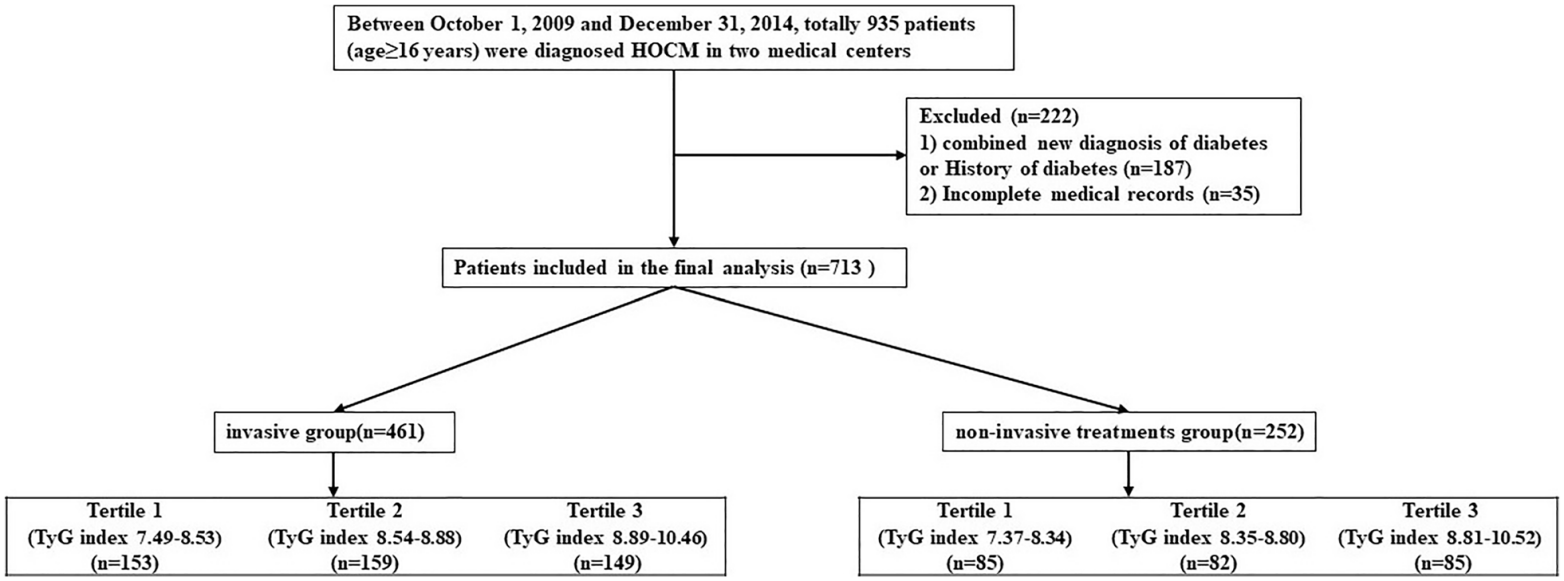
Flow diagram of patient selection

This study was a two-center, retrospective cohort study. All patients in this study were evaluated at two medical centers: the Peking University Third Hospital, and Fuwai Hospital (National Center of Cardiovascular Diseases, China). Between October 1, 2009, and December 31, 2014, a total of 935 patients (age ≥ 16 years) were diagnosed with HOCM. Among those participants, 713 subjects, non-combined diagnoses of diabetes, with complete information of clinical information, and medical history, congenital heart disease, cardiac valve disease, and amyloidosis, were selected. Considering that patients undergo invasive treatment (including surgical myectomy and alcohol septal ablation), the clinical symptoms and long-term prognosis of patients are significantly improved when the left ventricular outflow tract obstruction is lifted. We first divided the current study population into two groups receiving invasive (n = 461) and non-invasive treatments (n = 252). Subsequently, in each group, patients were further divided into three separate groups according to the level of the TyG index (Fig. 1).

#### Diagnosis of diabetes in this study

Diabetes mellitus is a chronic metabolic disorder characterized by hyperglycemia resulting from defects in insulin secretion, insulin action, or both. The diagnosis of diabetes in this study is based on the measurement of plasma glucose concentration. The American Diabetes Association (ADA) has recommended the following diagnostic criteria for diabetes [17]: HbA1c ≥ 6.5%. HbA1c is a measure of glycated hemoglobin, which reflects average blood glucose levels over the previous 2–3 months. Fasting plasma glucose (FPG) ≥ 126 mg/dL (7.0 mmol/L). Fasting is defined as no caloric intake for at least 8 h. 2-h plasma glucose (2-h PG) ≥ 200 mg/dL (11.1 mmol/L) during an oral glucose tolerance test (OGTT). The test should be performed as described by the World Health Organization, using a glucose load containing the equivalent of 75 g of anhydrous glucose dissolved in water. Random plasma glucose (RPG) ≥ 200 mg/dL (11.1 mmol/L) in a patient with classic symptoms of hyperglycemia or hyperglycemic crisis.

#### Diagnosis of HOCM

The diagnosis of HOCM was based on [18–20]: 1, wall thickness ≥ 15 mm in one or more LV myocardial segments, as measured by any imaging technique (echocardiography, cardiac magnetic resonance imaging, or computed tomography); 2, wall thickness (13–14 mm) with family history, non-cardiac symptoms and signs, electrocardiogram(ECG) abnormalities, laboratory tests, and muti-modality cardiac imaging; 3, patients with LV outflow tract obstruction (LVOTO) were based on: dynamic LVOT obstruction due to systolic anterior motion of the mitral valve, with an LVOT gradient ≥ 30 mmHg at rest or during physiological provocation such as Valsalva maneuver, standing, and exercise. Significant dynamic LVOT obstruction was documented by 2-dimensional and Doppler echocardiography or, in those cases where echocardiography was insufficient, by invasive hemodynamic catheterization with provocation.

#### Criteria for receiving invasive treatment

Invasive treatment to reduce LVOTO should be considered in patients with an LVOTO gradient ≥ 50 mmHg at rest or with provocation, moderate-to-severe symptoms (NYHA III-IV), and/or recurrent exertional syncope despite maximally tolerated drug therapy [20]. The choice of alcohol septal ablation or surgical myectomy was made through a shared decision-making process after a discussion of the risks of benefits of each alternative.

Surgical myectomy is considered as the gold standard therapy for HOCM. Excision of protruding septal muscle results in enlargement of the left ventricular outflow tract with a decrease in the severity or complete elimination of the left ventricular outflow tract obstruction. Transcatheter alcohol septal ablation is another preferred alternative treatment. Alcohol is injected directly into a septal branch of the anterior descending artery supplying the basal part of the septum through an interventional catheter to induce local myocardial necrosis, lead to scar formation and reduce left ventricular outflow tract obstruction.

#### Myocardial perfusion imaging (MPI)/Myocardial metabolic imaging (MMI)

We examined myocardial perfusion/metabolism in a subset of these HOCM patients. Myocardial perfusion/metabolic examination Myocardial perfusion examination was performed using a Philips dual probe SPECT/CT (Precedence16) scanner equipped with a low-energy high-resolution collimator from the Netherlands [21, 22]. The imaging agent was 99mTc-MIBI (Atomic High-Tech Co., Ltd., China), and 740–925 MBq of imaging agent was injected intravenously and visualized 1.0–1.5 h later. The patient's heart was included in the effective field of view, 25 s/frame, and a total of 32 frames were acquired. Resting myocardial perfusion tomography images were acquired with the dual probe rotated 180°, and reconstruction was performed by the Astonish iterative method. PET myocardial metabolic imaging was performed on alternate days, using Siemens PET/CT equipped with 52-ring crystal and 128-layer spiral CT, and the imaging agent was 18F-FDG (Atomic High Technology Co., Ltd., China), and different doses of insulin were injected according to the patient's blood glucose level, weight and waist circumference index, diabetes and lipid metabolism. When the blood glucose concentration reached 5.55–7.77 mmol/L, 3.7–5.55 MBq/kg of imaging agent was injected, and PET/CT images were acquired 60 min later. The patient's heart was included in the effective field of view at 25 s/frame, and a total of 32 frames were acquired with the probe rotated 360°. myocardial metabolic tomography images were acquired, and the original images were reconstructed in the short axis using TrueX and TOF Ultral HD iterative methods. the CT data were used to form attenuation-corrected images. For gated acquisition, myocardial perfusion/metabolism images were acquired at 8 frames per cardiac cycle. Myocardial perfusion/metabolism images were analyzed on a Siemens syngo multimodal processing workstation using Cedars QPS/QGS software for simultaneous post-processing, and three axial images (vertical long axis, horizontal long axis, and short axis) were reconstructed.

### Follow-up and endpoints

Follow-up started at the time of the patient's first clinic contact after October 1, 2009. At baseline, all patients were evaluated for the following characteristics: age, sex, NYHA functional class, maximum left ventricular wall thickness (LVWT), maximum (Provo cable) LVOT gradient, left ventricular function, atrial fibrillation, and conventional risk factors for cardiac death.

The primary endpoints of this study were Cardiogenic death during long-term follow-up. Mortality and adverse events were retrieved from hospital patient records at the center where follow-up occurred, from civil service population registers, and information provided by patients themselves and/or their general practitioners. Patients lost to follow-up were censored upon the last contact with them. If no events occurred during follow-up, the administrative censoring date was set for December 31, 2016.

### Data analysis

Statistical analysis was assessed with SPSS 27.0 statistical package for Windows, and R software version 4.2.2 (R Foundation for Statistical Computing). All continuous variables are presented as means ± SD and analysis of variance was used to compare means across multiple groups. The relationships between parametric variables were assessed by multiple linear regression analysis. Initial differences in baseline characteristics between achieved treatment groups were sought in bivariable analysis by using χ^2^ tests, Fisher exact tests, Student *t* tests, and Kruskal–Wallis analysis of variance. Cox proportional hazards model was used to estimate the hazard ratio (HR). Kaplan–Meier analysis was used to study the cumulative survival of different groups. A P-value less than 0.05 was considered statistically significant.

Restricted cubic spline (RCS) is a statistical method used to model nonlinear relationships between a continuous predictor variable and an outcome variable. It is an extension of the linear regression model and allows for flexible modeling of the relationship by using cubic polynomial functions. In our study, we use the RCS method provides a flexible and powerful way to model nonlinear relationships in R. We chose to use four knots, placed at the 10th, 50th, and 90th percentiles of the distribution of TyG-index in our study population, to allow for a flexible yet parsimonious modeling of the non-linear relationship between TyG-index and cardiovascular events. We assessed the overall fit of the model using the likelihood ratio test and examined the shape of the spline function using graphical displays.

### Data and resource availability statement

The datasets generated during and/or analyzed in the current study are available from the corresponding author upon reasonable request.

## Results

### Baseline characteristics of all patients in this study (Table 1)

The study population consisted of 713 HOCM patients without diabetes, with a mean age of 49.88 ± 13.04 years and a male predominance (59.5%). The mean BMI was 25.55 ± 6.0 kg/m^2^, and the mean systolic and diastolic blood pressures were 118.90 ± 18.44 mmHg and 72.64 ± 11.37 mmHg, respectively. The mean heart rate was 71.49 ± 10.17 bpm, and the mean NT-proBNP was 1910.23 ± 1743.37 fmol/mL. The mean serum creatinine was 76.30 ± 21.13 µmol/L, and the mean uric acid level was 319.16 ± 116.27 µmol/L. The mean blood potassium level was 4.16 ± 0.35 mmol/L, and the mean HGB was 122.34 ± 21.51 g/L. The mean h-CRP was 7.64 ± 6.27 mg/L, and the mean CKMB was 13.71 ± 6.99 ng/ml. The mean FFA was 0.43 ± 0.21 mmol/l, and the mean triglyceride level was 140.42 ± 76.13 mg/dl. The mean LDL was 2.31 ± 0.89 mmol/L, and the mean HDL was 0.96 ± 0.33 mmol/L. The mean HbA1c was 5.56 ± 0.42%, and the mean fasting blood glucose was 95.37 ± 14.29 mg/dl. The mean TyG index was 8.70 ± 0.47.

**Table 1:** Baseline characteristics of the study population

	Total (n = 713)
Demographics	
Age, year	49.88 ± 13.04
Male, n (%)	424 (59.5%)
TyG index	8.70 ± 0.47
BMI (kg/m^2^)	25.55 ± 6.0
Systolic BP (mmHg)	118.90 ± 18.44
Diastolic BP (mmHg)	72.64 ± 11.37
Heart Rate (bpm)	71.49 ± 10.17
NT-proBNP (fmol/mL)	1910.23 ± 1743.37
Cr (µmol/L)	76.30 ± 21.13
Uric acid(µmol/L)	319.16 ± 116.27
Blood potassium level (mmol/L)	4.16 ± 0.35
HGB(g/L)	122.34 ± 21.51
h-CRP(mg/L)	7.64 ± 6.27
CKMB(ng/ml)	13.71 ± 6.99
FFA(mmol/l)	0.43 ± 0.21
Triglyceride (mg/dl)	140.42 ± 76.13
LDL-C (mmol/L)	2.31 ± 0.89
HDL-C(mmol/L)	0.96 ± 0.33
Total cholesterol level (mmol/L)	3.96 ± 1.06
HbA1c (%)	5.56 ± 0.42
Fasting blood glucose(mg/dl)	95.37 ± 14.29
WBC(10^9/L)	8.12 ± 2.71
Comorbidities and risk factors	
Hypertension, n (%)	212 (29.7%)
Dyslipidemia, n (%)	159 (22.3%)
Atrial fibrillation, n (%)	91 (12.8%)
Non- sustained ventricular tachycardia, n (%)	19 (2.7%)
Smoking, n (%)	297 (41.7%)
NYHA Class III or IV, n (%)	90 (12.6%)
Echocardiography	
Interventricular septal thickness (mm)	19.97 ± 5.20
LV end-diastolic diameter (mm)	42.31 ± 5.97
LV posterior wall thickness (mm)	12.01 ± 2.97
LV ejection fraction (%)	67.85 ± 8.77
LV outflow tract gradient, at rest (mmHg)	74.24 ± 33.80
Inner diameter of ascending aorta (mm)	30.26 ± 9.25
Left atrial anteroposterior diameter (mm)	40.03 ± 13.12
Left ventricular posterior wall thickness (mm)	12.01 ± 2.97
Anterior posterior diameter of right ventricle (mm)	20.28 ± 4.67
Medications	
Beta-blocker, n (%)	364 (51.1%)
ACEI/ARB, n (%)	53 (7.4%)
Statin, n (%)	86 (12.1%)
Calcium antagonist, n (%)	107 (15.0%)

Values are mean ± SD or n (%)

*BMI* body mass index, *NT-proBNP* N-terminal pro-brain natriuretic peptide, *Cr* serum creatinine, *BP* blood pressure, *FFA* Free fat acid, *HbA1c* glycosylated hemoglobin, *TyG* *index*, Triglyceride-glucose index, *NYHA* New York Heart Association, *LV* left ventricle, *ACEI/ARB* angiotensin-converting enzyme inhibitor/angiotensin receptor blocker

Comorbidities and risk factors included hypertension (29.7%), dyslipidemia (22.3%), atrial fibrillation (12.8%), non-sustained ventricular tachycardia (2.7%), smoking (41.7%), and NYHA Class III or IV (12.6%).

Echocardiographic measurements included interventricular septal thickness (19.97 ± 5.20 mm), LV end-diastolic diameter (42.31 ± 5.97 mm), LV posterior wall thickness (12.01 ± 2.97 mm), LV ejection fraction (67.85 ± 8.77%), and LV outflow tract gradient at rest (74.24 ± 33.80 mmHg).

In terms of medications, 51.1% of patients were treated with beta-blockers, 7.4% with ACEI/ARB, 12.1% with statins, and 15.0% with calcium antagonists. Regarding procedures, 51.3% of patients underwent septal myectomy and 13.3% underwent alcohol septal ablation.

### Baseline characteristics of patients in the invasive treatment group (Table 2)

The data provided earlier is a table of baseline clinical characteristics of participants in the invasive treatment group of a study. The invasive treatment group is composed of 461 individuals who were divided into tertiles (three equal groups) based on their TyG index (Tertile 1: TyG index 8.09 ± 0.24, n = 153; Tertile 2: TyG index 8.71 ± 0.22, n = 159; Tertile 3: TyG index 9.48 ± 0.48, n = 149;).

**Table 2:** Baseline characteristics of the Invasive treatment group and Non-invasive treatment group

	Invasive treatment group					Non-invasive treatment group				
	Total	Tertile 1	Tertile 2	Tertile 3	P	Total	Tertile 1	Tertile 2	Tertile 3	P
	(n = 461)	(n = 153)	(n = 159)	(n = 149)		(n = 252)	(n = 85)	(n = 82)	(n = 85)	
Demographics										
TyG index	8.74 ± 0.42	8.29 ± 0.21	8.74 ± 0.10	9.19 ± 0.28	< 0.001	8.62 ± 0.53	8.09 ± 0.24	8.57 ± 0.14	9.21 ± 0.35	< 0.001
Age, year	47.00 ± 11.72	45.54 ± 13.02	46.35 ± 11.14	49.19 ± 10.64	0.017	55.13 ± 13.69	55.84 ± 15.71	55.14 ± 14.29	54.43 ± 10.75	0.799
Male, n (%)	274 (59.4%)	89 (58.2%)	92 (57.9%)	93 (62.4%)	0.666	150(59.5%)	44(51.8%)	47(57.3%)	59 (69.4%)	0.057
BMI (kg/m^2^)	25.35 ± 4.15	24.28 ± 4.25	25.42 ± 4.47	26.38 ± 3.34	< 0.001	25.95 ± 8.59	23.55 ± 3.99	26.98 ± 13.35	27.40 ± 4.78	0.012
Systolic BP (mmHg)	115.10 ± 15.92	111.81 ± 14.93	114.56 ± 16.40	119.15 ± 15.61	< 0.001	125.88 ± 20.63	125.35 ± 20.58	122.48 ± 16.98	129.64 ± 23.29	0.08
Diastolic BP (mmHg)	70.86 ± 10.93	69.57 ± 11.73	70.16 ± 10.63	72.99 ± 10.11	0.016	75.92 ± 11.46	74.73 ± 11.82	75.65 ± 11.36	77.37 ± 11.17	0.317
Heart Rate (bpm)	72.38 ± 9.52	72.12 ± 9.05	71.88 ± 9.45	73.18 ± 10.06	0.456	69.86 ± 11.10	70.33 ± 12.22	70.50 ± 11.30	68.77 ± 9.69	0.545
NT-proBNP (fmol/mL)	2015.84 ± 1780.52	2167.99 ± 2052.93	1965.20 ± 1622.10	1907.36 ± 1625.27	0.514	1732.80 ± 1668.46	2176. 45 ± 1889.25	1630.29 ± 1523.99	1393.66 ± 1486.57	0.514
Cr (µmol/L)	76.58 ± 21.21	72.63 ± 18.21	75.03 ± 20.71	82.31 ± 23.42	< 0.001	75.79 ± 21.00	70.34 ± 17.89	74.04 ± 15.81	82.91 ± 25.93	< 0.001
Uric acid(µmol/L)	293.64 ± 113.02	287.03 ± 112.95	278.42 ± 100.13	316.66 ± 122.75	0.008	365.85 ± 107.48	339.17 ± 125.87	359.01 ± 81.35	399.14 ± 102.02	0.001
Blood potassium level (mmol/L)	4.19 ± 0.35	4.15 ± 0.32	4.17 ± 0.35	4.26 ± 0.38	0.012	4.11 ± 0.35	4.11 ± 0.39	4.11 ± 0.33	4.10 ± 0.33	0.958
HGB(g/L)	116.48 ± 20.53	116.29 ± 21.59	115.14 ± 20.35	118.11 ± 19.60	0.444	133.08 ± 19.02	130.20 ± 19.05	133.29 ± 17.91	135.76 ± 19.82	0.161
h-CRP(mg/L)	9.84 ± 6.03	8.31 ± 6.44	10.49 ± 5.78	10.72 ± 5.56	0.001	3.62 ± 4.45	3.89 ± 4.86	3.19 ± 4.27	3.77 ± 4.20	0.561
CKMB(ng/ml)	14.25 ± 7.41	13.65 ± 7.49	14.62 ± 7.69	14.49 ± 7.04	0.46	12.70 ± 6.01	11.78 ± 4.83	13.66 ± 6.58	12.70 ± 6.01	0.129
FFA(mmol/l)	0.43 ± 0.21	0.40 + 0.23	0.43 ± 0.20	0.45 ± 0.20	0.26	0.45 ± 0.21	0.45 ± 0.24	0.40 ± 0.16	0.50 ± 0.22	0.171
Triglyceride (mg/dl)	139.17 ± 69.20	89.38 ± 17.41	128.52 ± 20.75	201.67 ± 86.47	< 0.001	142.73 ± 87.58	78.248 ± 16.13	121.61 ± 19.18	227.58 ± 101.85	< 0.001
LDL-C (mmol/L)	2.10 ± 0.84	2.02 ± 0.90	2.07 ± 0.77	2.22 ± 0.85	0.088	2.70 ± 0.83	2.39 ± 0.69	2.90 ± 0.81	2.81 ± 0.90	< 0.001
HDL-C(mmol/L)	0.89 ± 0.32	1.00 ± 0.34	0.87 ± 0.29	0.81 ± 0.29	< 0.001	1.10 ± 0.31	1.19 ± 0.37	1.15 ± 0.28	0.95 ± 0.23	< 0.001
HbA1c (%)	5.50 ± 0.41	5.40 ± 0.34	5.49 ± 0.43	5.64 ± 0.45	0.001	5.64 ± 0.42	5.53 ± 0.51	5.62 ± 0.32	5.80 ± 0.35	0.002
Fasting blood glucose(mg/dl)	98.48 ± 14.42	91.89 ± 13.32	99.06 ± 12.66	104.64 ± 14.48	< 0.001	89.68 ± 12.16	85.86 ± 10.75	87.89 ± 9.85	95.21 ± 13.57	< 0.001
Comorbidities and risk factors										
Hypertension, n (%)	100 (21.7%)	27 (17.8%)	34(21.4%)	39(26.4%)	0.195	112 (44.6%)	32 (38.1%)	31 (37.8%)	49 (57.6%)	0.012
Dyslipidemia, n (%)	68 (14.8%)	15 (9.9%)	21(13.2%)	32(21.5%)	0.014	91 (36.3%)	21 (25.0%)	25 (30.5%)	45 (52.9%)	< 0.001
Atrial fibrillation, n (%)	44 (9.5%)	17 (11.1%)	13(8.2%)	14(9.4%)	0.676	47 (18.7%)	18 (21.2%)	12 (14.6%)	17 (20.0%)	0.514
Non- sustained ventricular tachycardia, n (%)	6 (1.3%)	2 (1.3%)	3(1.9)	1(0.7%)	0.642	13 (5.2%)	4 (4.7%)	4 (4.9%)	5 (5.9%)	0.932
Smoking, n (%)	187 (40.6%)	57 (37.3%)	64(40.3%)	66(44.9)	0.399	110 (44.0%)	29 (34.1%)	40 (49.4%)	41 (48.8%)	0.078
NYHA Class III or IV, n (%)	60 (13%)	19 (29.7%)	19(38%)	22(43.1)	0.316	30 (39.5%)	14 (41.2%)	6 (28.6%)	10 (47.6%)	0.434
Echocardiography										
Interventricular septal thickness (mm)	20.02 ± 5.13	20.52 ± 5.32	20.08 ± 4.97	19.42 ± 5.06	0.171	19.88 ± 5.34	19.87 ± 5.12	20.28 ± 6.18	19.51 ± 4.70	0.659
LV end-diastolic diameter (mm)	42.32 ± 5.72	42.01 ± 6.23	42.41 ± 5.61	42.53 ± 5.29	0.703	42.30 ± 6.42	42.05 ± 6.36	41.89 ± 6.14	42.93 ± 6.75	0.533
LV posterior wall thickness (mm)	12.06 ± 2.84	12.20 ± 3.24	11.85 ± 2.47	12.15 ± 2.75	0.502	11.91 ± 3.20	11.60 ± 3.39	11.76 ± 3.22	12.38 ± 2.97	0.255
LV ejection fraction (%)	67.47 ± 8.73	68.18 ± 7.84	67.04 ± 9.36	67.21 ± 8.93	0.47	68.56 ± 8.82	68.36 ± 10.49	68.83 ± 7.69	68.49 ± 8.06	0.47
LV outflow tract gradient, at rest (mmHg)	79.61 ± 32.70	82.42 ± 34.30	76.73 ± 30.23	79.73 ± 33.47	0.313	64.25 ± 33.59	65.77 ± 35.63	60.90 ± 31.40	65.86 ± 33.68	0.568
Medications										
Beta-blocker, n (%)	226 (49.0%)	80 (74.8%)	67 (64.4%)	79 (78.2%)	0.07	138 (83.1%)	39 (84.8%)	46 (86.8%)	53 (79.1%)	0.504
ACEI/ARB, n (%)	19 (4.1%)	8 (7.5%)	5 (4.9)	6 (5.9)	0.737	34 (20.7%)	5 (10.9%)	8 (15.1%)	21 (20.7%)	0.011
Statin, n (%)	34 (7.4%)	14 (13.1)	11 (10.8%)	9 (9.0%)	0.641	53 (31.5%)	11 (23.9%)	13 (24.5%)	28 (42.4%)	0.048
Calcium antagonist, n (%)	39 (8.5%)	16 (15%)	14 (13.7%)	9 (8.9%)	0.386	68 (41.1%)	16 (34.8%)	19 (35.8%)	33 (50.0%)	0.172

Values are mean ± SD or n (%)

*BMI* body mass index, *NT-proBNP* N-terminal pro-brain natriuretic peptide, *Cr* serum creatinine, *BP* blood pressure, *FFA* Free fat acid, *HbA1c* glycosylated hemoglobin, *TyG* index, Triglyceride-glucose index, *NYHA* New York Heart Association, *LV* left ventricle, *ACEI/ARB* angiotensin-converting enzyme inhibitor/angiotensin receptor blocker

The table contains information about demographic factors such as age, gender, body mass index (BMI), blood pressure, heart rate, and laboratory measurements such as serum levels of NT-proBNP (a biomarker of heart failure), creatinine (a measure of kidney function), uric acid, blood potassium, hemoglobin (Hb), high-sensitivity C-reactive protein (h-CRP), creatine kinase-MB (CKMB, a biomarker of heart damage), free fatty acids (FFA), triglycerides, low-density lipoprotein (LDL) cholesterol, high-density lipoprotein (HDL) cholesterol, hemoglobin A1c (HbA1c), and fasting blood glucose.

The table also shows the prevalence of various comorbidities and risk factors such as hypertension, dyslipidemia, atrial fibrillation, non-sustained ventricular tachycardia, and smoking among the participants. The p-value column indicates the statistical significance of differences in each variable between the tertiles of the TyG index.

The P-values shown in the table indicate the significance level of the differences in the baseline characteristics of the study population between tertiles. For instance, a P-value of < 0.001 for the TyG index means that the mean TyG index values in tertiles 1, 2, and 3 were significantly different from each other. Similarly, the P-value of < 0.001 for BMI means that the mean BMI values in tertiles 1, 2, and 3 were significantly different from each other.

The P-values for age (0.017), diastolic blood pressure (0.016), uric acid (0.008), blood potassium level (0.012), h-CRP (0.001), fasting blood glucose (< 0.001), dyslipidemia (0.014), and HDL (0.001) were statistically significant, indicating that there were significant differences in these characteristics between the tertiles.

On the other hand, the P-values for gender (0.666), heart rate (0.456), NT-proBNP (0.514), CKMB (0.46), FFA (0.26), LDL (0.088), hypertension (0.195), atrial fibrillation (0.676), and non-sustained ventricular tachycardia (0.642) were not statistically significant. It means that the differences in these characteristics between the tertiles were not significant.

### Baseline characteristics of patients in the non-invasive treatment group (Table 2)

Based on the statistical analysis results for the baseline table of the non-invasive treatment group (Table 3), the mean value of the TyG index was significantly different among the three tertiles (Tertile 1: TyG index 8.09 ± 0.24, n = 85; Tertile 2: TyG index 8.71 ± 0.22, n = 82; Tertile 3: TyG index 9.48 ± 0.48, n = 85) with a p-value less than 0.001. This suggests that there are significant differences in insulin resistance levels among the three tertiles. Additionally, the mean age and BMI were also significantly different among the tertiles (p < 0.001 for both), indicating that there are differences in age and body mass index across the groups as well.

**Table 3:** Correlations between the TyG index and risk factors of HOCM

Variables	Invasive treatment group		Non-invasive treatment group	
	Correlation coefficient (r)	p-value	Correlation coefficient (r)	p-value
LV outflow tract gradient, at rest (mmHg)	− 0.072	0.483	− 0.099	0.521
LV ejection fraction (%)	− 0.220	**0.031**	0.072	0.644
Interventricular septal thickness (mm)	− 0.208	**0.042**	− 0.127	0.411
LV end-diastolic diameter (mm)	0.262	**0.010**	0.377	**0.012**
LV posterior wall thickness (mm)	0.073	0.478	− 0.096	0.534
Systolic BP (mmHg)	0.114	0.267	− 0.098	0.528
Diastolic BP (mmHg)	0.105	0.308	− 0.028	0.858
Heart Rate (bpm)	− 0.024	0.820	− 0.081	0.599
NYHA Class III or IV, n (%)	− 0.038	0.710	− 0.064	0.678
Atrial fibrillation, n (%)	− 0.015	0.885	− 0.015	0.920
LDL-C (mmol/L)	− 0.014	0.895	0.400	**0.007**
HDL-C(mmol/L)	− 0.425	** < 0.001**	− 0.382	**0.010**
Cr (µmol/L)	0.250	**0.014**	0.207	0.177
h-CRP(mg/L)	0.227	**0.026**	0.100	0.518
NT-proBNP (fmol/mL)	− 0.224	**0.028**	− 0.224	**0.028**
Age	0.099	**0.033**	− 0.028	0.661
BMI	0.336	** < 0.001**	0.168	0.271

*TyG* index triglyceride-glucose index, *BMI* body mass index, *LVEF* left ventricle ejection fraction, *LDL-C* low-density lipoprotein-cholesterol, *HDL-C* high-density lipoprotein-cholesterol

p-values in bold are < 0.05

Specifically, patients in tertile 1 have the lowest TyG index values, BMI, triglyceride, LDL, and HbA1c levels, while having the highest HDL and fasting blood glucose levels. Conversely, patients in tertile 3 have the highest TyG index values, BMI, triglyceride, and LDL levels, while having the lowest HDL and fasting blood glucose levels. There are also statistically significant differences among the three tertiles for some comorbidities and risk factors. Patients in tertile 3 have a higher prevalence of hypertension and dyslipidemia compared to those in Tertile 1 and 2.

### Correlations between the TyG index and risk factors (Table)

The associations between the TyG index and HOCM risk factors were examined using Spearman correlation analysis. As shown in Table 4, in the invasive treatment group, TyG index was positively associated with LV end-diastolic diameter, Cr, h-CRP, Age, and BMI (p < 0.05). Negatively associated with LV ejection fraction, Interventricular septal thickness, HDL-C, NT-proBNP (p < 0.05). No significant correlation was observed between LV outflow tract gradient, LV posterior wall thickness, Systolic BP, Diastolic BP, Heart Rate, NYHA Class III or IV, Atrial fibrillation, and LDL-C (Table 3).

**Table 4:** Univariate/multivariate cox analysis for cardiovascular events in the invasive treatment group

	Invasive treatment group					Non-invasive treatment group			
	Univariate		Multivariate			Univariate		Multivariate	
	HR (95% CI)	P	HR (95% CI)	P		HR (95% CI)	P	HR (95% CI)	P
Age, year	0.994 (0.953, 1.038)	0.799	–	–	BMI	0.889 (0.796, 0.993)	**0.037**	0.948 (0.851,1.055)	0.327
BMI	0.945 (0.816, 1.095)	0.452	1.007 (0.892, 1.136)	0.913	Cr (µmol/L)	1.017 (1.006, 1.028)	**0.002**	1.012 (0.998, 1.025)	0.089
Systolic BP (mmHg)	0.963 (0.930, 0.997)	**0.031**	0.978 (0.942,1.016)	0.255	Uric acid (µmol/L)	1.006 (1.003, 1.009)	** < 0.001**	1.004 (1.002,1.007)	**0.002**
Diastolic BP (mmHg)	0.951 (0.903, 1.002)	0.059	–	–	Triglyceride (mg/dl)	0.990 (0.982,0.999)	**0.022**	**–**	**–**
Cr (µmol/L)	1.008 (0.989, 1.028)	0.407	–	–	LDL-C (mmol/L)	0.807 (0.501,1.301)	0.38	–	–
Uric acid (µmol/L)	1.002 (1.000, 1.005)	0.091	–	–	HDL-C (mmol/L)	0.175 (0.038,0.806)	**0.025**	0.144 (0.032,0.654)	**0.012**
Blood potassium level (mmol/L)	1.325 (0.364,4.818)	0.669	–	–	HbA1c (%)	2.430 (0.668,8.847)	0.178	–	–
h-CRP(mg/L)	0.932 (0.861, 1.008)	0.078	**–**	**–**	Fasting blood glucose(mg/dl)	0.977 (0.943,1.012)	0.198	–	–
Triglyceride (mg/dl)	0.996 (0.986,1.005)	0.366	–	–	Hypertension	0.754 (0.338,1.678)	0.488	–	–
HDL(mmol/L)	1.556 (0.341,7.113)	0.568	–	–	Dyslipidemia	0.649 (0.279,1.505)	0.314	–	–
HbA1c (%)	1.441 (0.230,9.046)	0.697	–	–	TyG index	0.393 (0.177,0.873)	**0.022**	0.179 (0.063, 0.508)	**0.001**
Fasting blood glucose(mg/dl)	0.979 (0.944,1.015)	0.252	–	–					
Dyslipidemia	1.209 (0.344,4.250)	0.768	–	–					
TyG index	0.249 (0.079,0.791)	**0.018**	0.215 (0.051, 0.902)	**0.036**					

Abbreviations are listed in and. p-values in bold are  < 0.05

As shown in Table 4, in the non-invasive treatment group, the TyG index was positively associated with LV end-diastolic diameter, and LDL-C (p < 0.05). Negatively association was observed between TyG and HDL-C(mmol/L), NT-proBNP (fmol/mL). No significant correlation was observed between TyG and LV outflow tract gradient, LV ejection fraction, Interventricular septal thickness, LV posterior wall thickness, Systolic BP, Diastolic BP, NYHA Class III or IV, Atrial fibrillation, Cr, h-CRP, Age, BMI.

### TyG index and cardiovascular events (Fig. 2, Table 4)

**Fig. 2:**
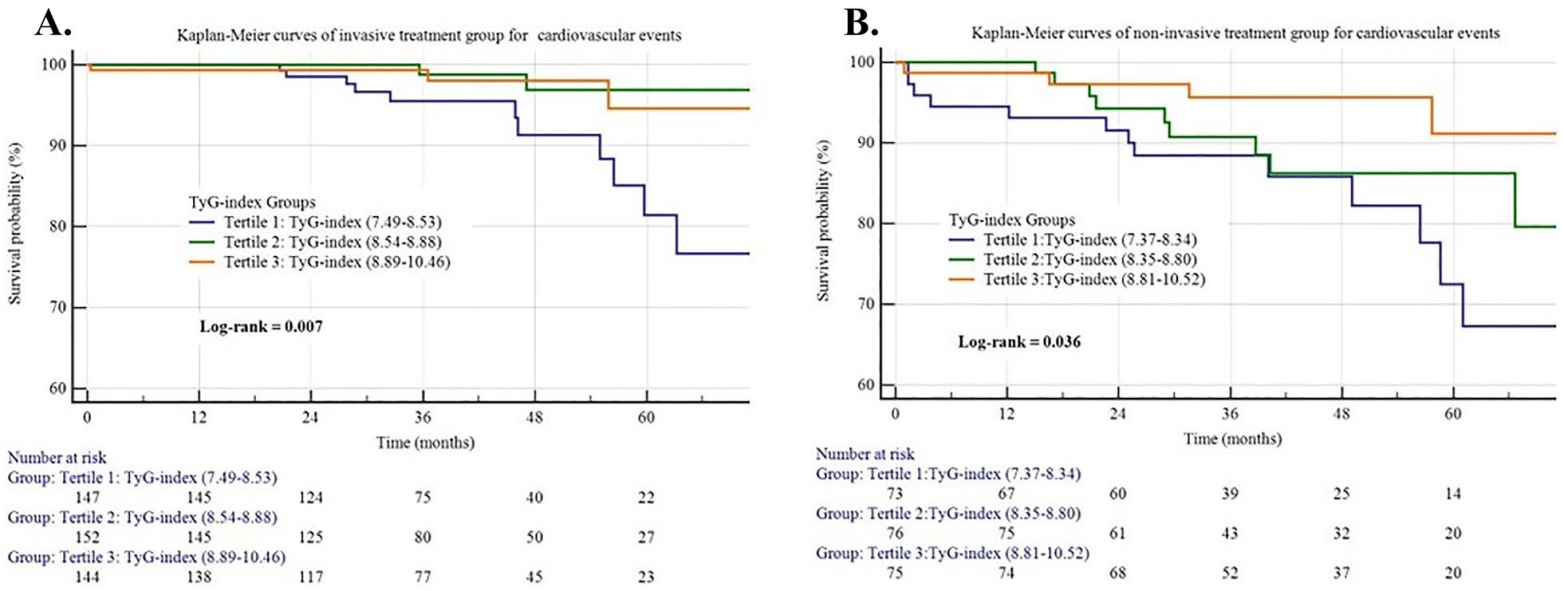
Kaplan–Meier survival curve of invasive treatment group and non-invasive treatment group for cardiovascular events across the TyG index tertiles

The follow-up time of this study was 41.47 ± 17.63 months. During the follow-up, in the invasive treatment group, 16(3.5%) cardiovascular events were recorded. To show the outcomes of patients with different levels of the TyG index, we generated Kaplan–Meier survival plots (Fig. 2). As shown in Fig. 2, the cumulative incidence of cardiovascular events decreased incrementally across tertiles of the TyG index (log-rank test, p = 0.007). Univariate Cox regression analysis was used to identify the factors associated with cardiovascular events. Systolic BP and the TyG index were found to be protective factors for cardiovascular events. The unadjusted HR (95% CI) for risk of cardiovascular events with per SD increase in the TyG index was 0.249 (95%CI 0.079–0.791, P = 0.018). Multivariate Cox proportional hazards regression analysis showed that the TyG index remained significant after adjusting for confounders (HR = 0.215, 95%CI 0.051–0.902, P = 0.036).

In the non-invasive treatment group, 26(10.3%) cardiovascular events were recorded. To show the outcomes of patients with different levels of the TyG index, we also generated Kaplan–Meier survival plots (Fig. 2). As shown in Fig. 2, the cumulative incidence of cardiovascular events decreased incrementally across tertiles of the TyG index (log-rank test, p = 0.036). Univariate Cox regression analysis was used to identify the factors associated with cardiovascular events. As presented in Table 4, Cr, Uric acid was found to be a risk factor for cardiovascular events. BMI, Triglyceride, HDL-C, and the TyG index were found to be protective factors for cardiovascular events. The unadjusted HR (95% CI) for risk of cardiovascular events with per SD increase in the TyG index was 0.393 (95%CI 0.177–0.873, P = 0.022) (Table 4). Multivariate Cox proportional hazards regression analysis showed that the TyG index remained significant after adjusting for confounders (HR = 0.179, 95%CI 0.063–0.508, P = 0.001).

### Nonlinear relationship between tyg index and cardiovascular events: a restricted cubic spline (RCS) analysis (Fig. 3)

**Fig. 3:**
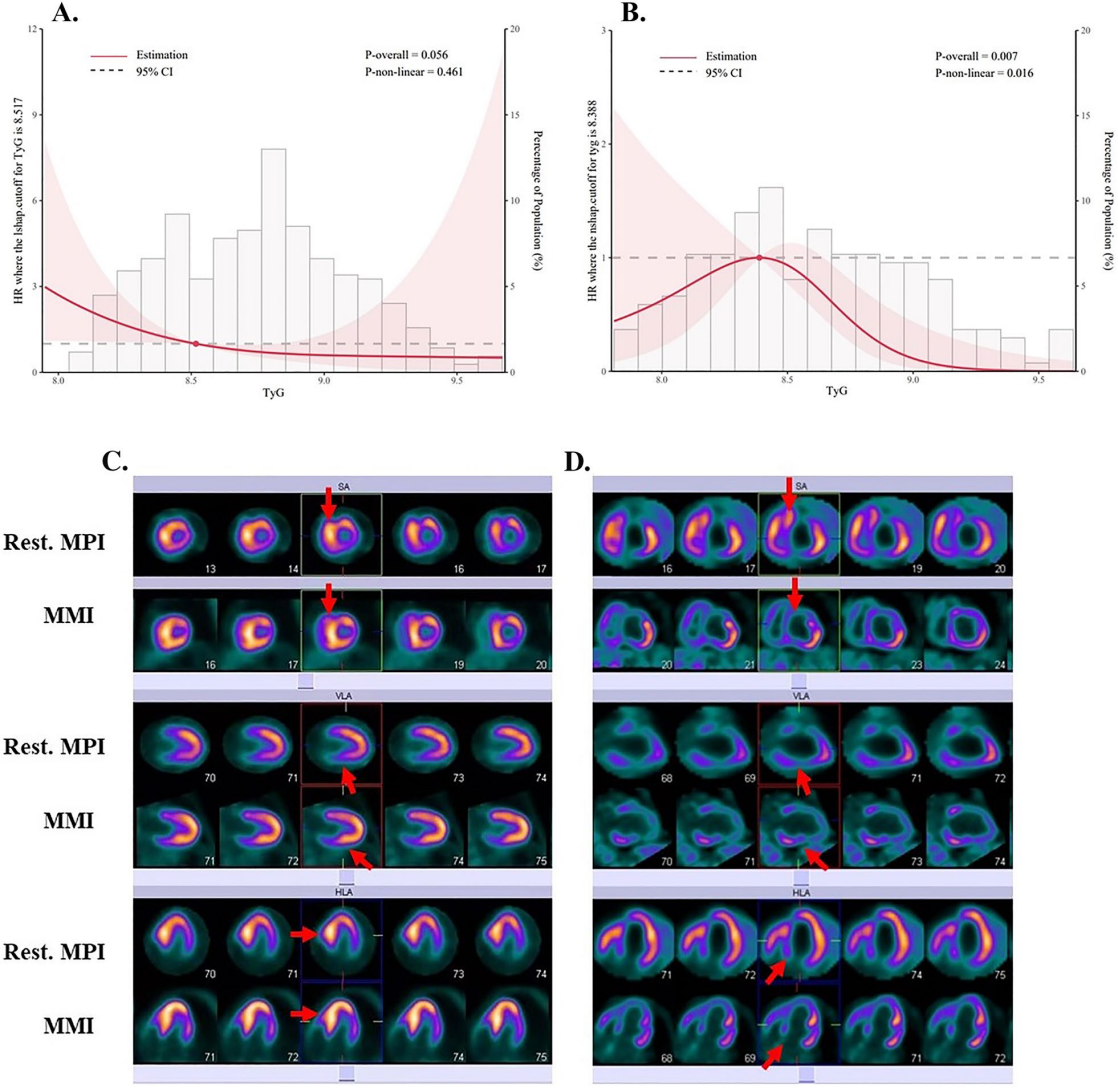
Restricted cubic spline models representing the associations between TyG index and the risk of cardiovascular events in HOCM with invasive treatment (**A**) and non-invasive treatment (**B**). Myocardial fusion imaging (MPl)/Myocardial metabolic imaging (MMl) of HOCM patient (**C**) and negative control patient (**D**)

We used restricted cubic splines to model the relationship between the TyG index and the risk of cardiovascular events in HOCM. We chose to use four knots, placed at the 10th, 50th, and 90th percentiles of the distribution of the TyG index in our study population, to allow for flexible yet parsimonious modeling of the non-linear relationship between the TyG index and cardiovascular events. We assessed the overall fit of the model using the likelihood ratio test and examined the shape of the spline function using graphical displays.

Figure 3A shows the estimated spline function for the relationship between the TyG index and the risk of cardiovascular events in HOCM with invasive treatment, based on our restricted cubic spline model. The results indicated “L-shaped” associations of the invasive treatment group with cardiovascular events. In this RCS analysis were adjusted Systolic BP and BMI. The curves showed a decreasing trend in the risk of cardiovascular events as the TyG index increased, although not statistically significant (p = 0.056).

Figure 3B shows the estimated spline function for the relationship between the TyG index and the risk of cardiovascular events in HOCM with non-invasive treatment, based on our restricted cubic spline model. The results indicated ‘‘n-shaped’’ associations of the non-invasive treatment group with cardiovascular events. In this RCS analysis were adjusted for BMI, Cr, Uric acid, and HDL. The curves showed a decreasing trend in the risk of cardiovascular events as the TyG index increased (p = 0.007). This trend is particularly evident for TyG indices greater than 8.388.

### Myocardial perfusion imaging (MPI)/myocardial metabolic imaging (MMI) (Figure 3A)

We examined Myocardial perfusion imaging (MPI)/Myocardial metabolic imaging (MMI) in some patients with confirmed HOCM and also performed a comparative analysis with images of normal Myocardial perfusion imaging (MPI)/ Myocardial metabolic imaging (MMI). As shown in the pictures, both blood perfusion and glucose metabolism in the myocardium, in the septal area, anterior left ventricular wall, and apical region are significantly enhanced in HOCM patients compared to normal subjects.

## Discussion

Our study found that the TyG index was associated with cardiovascular events in both the invasive and non-invasive treatment groups. In the invasive treatment group, the cumulative incidence of cardiovascular events decreased as the TyG index decreased, and the TyG index was found to be a protective factor for cardiovascular events in both univariate and multivariate Cox regression analyses, even after adjusting for confounders. In the non-invasive treatment group, a similar pattern was observed, with the cumulative incidence of cardiovascular events decreasing as the TyG index decreased, and the TyG index was also found to be a protective factor for cardiovascular events in both univariate and multivariate Cox regression analyses, even after adjusting for confounders. In both groups, the TyG index was positively associated with LV end-diastolic diameter and negatively associated with LV ejection fraction. In the invasive treatment group, the TyG index was also positively associated with Cr, h-CRP, Age, and BMI, while negatively associated with Interventricular septal thickness and HDL-C, NT-proBNP. However, in the non-invasive treatment group, the TyG index was positively associated with LDL-C and negatively associated with HDL-C, and NT-proBNP.

This study used a restricted cubic spline model to explore the non-linear relationship between the TyG index and the risk of cardiovascular events in patients with HOCM. We found an ‘‘L-shaped’’ association between invasive treatment and cardiovascular events, and an ‘‘n-shaped’’ association between non-invasive treatment and cardiovascular events. The curves showed a decreasing trend in the risk of cardiovascular events as the TyG index increased. In Figure 3A, the estimated spline function for the relationship between the TyG index and the risk of cardiovascular events in HOCM with invasive treatment shows a decreasing trend in the risk of cardiovascular events as the TyG index increases, although not statistically significant (p = 0.056). The curve shows an ‘‘L-shaped’’ association, indicating that there may be a threshold below which the risk of cardiovascular events is relatively high, but above which the risk decreases more gradually. This analysis was adjusted for Systolic BP and BMI. In Figure 3B, the estimated spline function for the relationship between the TyG index and the risk of cardiovascular events in HOCM with non-invasive treatment shows a decreasing trend in the risk of cardiovascular events as the TyG index increases (p = 0.007). The curve shows an "n-shaped" association, indicating that the risk of cardiovascular events decreases rapidly up to a certain point, and then more gradually beyond that point. This trend is particularly evident for TyG indices greater than 8.388. This analysis was adjusted for BMI, Cr, Uric acid, and HDL. These results suggest that the relationship between the TyG index and the risk of cardiovascular events in HOCM may be non-linear and that there may be a threshold effect at which the risk of events decreases more rapidly as the TyG index increases. These findings may have implications for risk stratification and management of HOCM patients.

The Myocardial perfusion/Myocardial metabolic imaging of patients with hypertrophic obstructive cardiomyopathy also showed that glucose metabolism was significantly enhanced in the energy metabolism of patients with hypertrophic obstructive cardiomyopathy. Some previous studies have shown that patients with HOCM have alterations in myocardial energy metabolism, with a shift towards increased glucose metabolism [12, 23, 24]. Myocardial perfusion/metabolism imaging studies using positron emission tomography (PET) have demonstrated increased glucose uptake in the hypertrophied myocardium of patients with HOCM, indicating a shift towards glucose metabolism [11, 12].

In hypertrophic cardiomyopathy (HCM), the increased left ventricular pressure load can lead to alterations in myocardial energy metabolism. The energy substrate preference of the myocardium may shift from fatty acid oxidation to glucose oxidation, and this metabolic remodeling may have a protective effect on the damaged myocardium [25]. Studies have shown that glucose uptake and utilization are increased in the hypertrophied myocardium of HCM patients [11]. This increased glucose utilization is mediated by several factors, including activation of the insulin signaling pathway, upregulation of glucose transporters, and increased expression of glycolytic enzymes [12]. However, despite this shift towards glucose metabolism, HCM patients may still experience energy deficits due to impaired mitochondrial function and reduced oxidative phosphorylation capacity [26]. This impaired mitochondrial function may be due to genetic mutations in the mitochondrial DNA or nuclear genes that encode mitochondrial proteins, as well as other factors such as oxidative stress and inflammation.

Although there are differences in the functional alterations caused by different myosin genetic mutations, there is a consensus that they all lead to increased myosin Ca2 + sensitivity and impaired myosin energy metabolism. The various metabolic alterations in the myocardium of HCM patients are not independent but are organically linked. Mutations cause altered Ca2 + sensitivity and exhibit impaired energy metabolism. This metabolic impairment prevents the return of Ca2 + to the sarcoplasmic reticulum, disrupting Ca2 + and Na + cycling and triggering mitochondrial dysfunction. Impaired mitochondria further exacerbate the energy metabolism disorder and further trigger increased Ca2 + sensitivity and metabolic remodeling [25, 26]. The search for the link between the metabolic mechanisms of HCM and specific clinical manifestations is one of the current hot spots in basic research on HCM. There are also stage-specific differences in myocardial metabolic architecture in HCM patients. In early-stage HCM patients, symptoms are not obvious, cardiac function indices on routine noninvasive tests are within normal limits [27], metabolic substrates are generally not altered or undergo only minor metabolic remodeling during this period, and energy flow is not significantly reduced [28, 29]. In contrast, in the late stage of HCM, when ventricular dilatation and heart failure occur, myocardial remodeling intensifies due to the continuous chronic stimulation of various pathological factors, resulting in reduced ventricular compliance, restricted ventricular filling, increased end-diastolic pressure, severe diastolic dysfunction, and sometimes even systolic dysfunction [27]. Severe metabolic remodeling, reduced respiratory chain activity, and impaired mitochondrial oxidative energy fluxes also occur during this period [28, 29].

The lack of myocardial energy metabolism may aggravate myocardial fibrosis in HOCM. Studies have shown that metabolic alterations, including a shift from fatty acid to glucose metabolism, are characteristic of HOCM, and this metabolic remodeling may have a protective effect on the myocardium [30]. Insufficient glucose utilization and impairment of energy metabolism in HOCM may lead to the accumulation of toxic metabolites, increased oxidative stress, and activation of fibrogenesis pathways, which can contribute to the development and progression of myocardial fibrosis [26, 31]. Myocardial fibrosis, in turn, can lead to further impairment of myocardial energy metabolism and exacerbate cardiac dysfunction in HOCM [31].

LVOTO is a key pathophysiological feature of HOCM, and its alleviation through surgical or medical interventions has been shown to improve symptoms and prognosis in some patients. So, whether the improvement of left ventricular outflow tract obstruction (LVOTO) in patients with hypertrophic obstructive cardiomyopathy (HOCM) can lead to improvement in myocardial energy metabolism. This issue needs further research and exploration.

There is limited information available regarding the effect of targeted drugs for hypertrophic cardiomyopathy (HCM) on myocardial energy metabolism. Mavacamten and aficamten are small-molecule inhibitors of cardiac myosin that have been recently approved by the US Food and Drug Administration (FDA) for the treatment of obstructive HCM. These drugs work by reducing the hypercontractility of the heart muscle, which leads to a reduction in the pressure gradient across the left ventricular outflow tract (LVOT) and an improvement in symptoms. For example, Mavacamten is a novel small-molecule myocardial-specific myosin aliasing modulator that selectively inhibits myocardial myosin ATPase and reduces Na + , thereby reducing myocardial energy expenditure in patients with HCM, resulting in improved hypercontraction and diastolic abnormalities [32, 33]. However, further research is needed to confirm these findings and to determine the clinical significance of these effects in humans. On the other hand, we must also see that the failure of clinical trials of the metabolic remodeling drug trimetazidine (TMZ) [34] and the late sodium channel inhibitor ranolazine [35] suggests that the metabolic mechanisms of HCM are much more complex than is currently recognized.

## Conclusion

Our study suggests that alterations in myocardial energy metabolism may have an impact on disease progression and the development of adverse clinical outcomes in hypertrophic obstructive cardiomyopathy. The triglyceride glucose (TyG) index, a marker of insulin resistance, has been shown to be associated with adverse clinical outcomes in some patients with cardiovascular disease. In these studies, TyG was considered a risk factor for clinical outcomes in patients with coronary heart disease as well as heart failure with low ejection fraction [14, 15, 36–39]. There are no similar studies on the role of the triglyceride glucose (TyG) index and the prognostic impact of hypertrophic cardiomyopathy. In our study, we found that in patients with HOCM without comorbid diabetes, the long-term prognosis was also better in patients with elevated TyG, regardless of whether these patients received invasive therapeutic measures. The possible reason for our findings, which differ from the role of TyG in other cardiovascular diseases, is due to the unique hemodynamic mechanisms of hypertrophic obstructive cardiomyopathy. Restricted cubic spline plots also suggest a nonlinear association between TyG and cardiovascular events. Within a certain range, a state of elevated TyG may allow more glucose to be available for myocardial metabolism, which may have a degree of protective effect on myocardial remodeling and fibrosis, which in turn may affect the long-term prognosis of patients. Conversely, deficiencies in metabolic substrates may exacerbate the myocardial injury and lead to a worsening prognosis in patients with HCM.

Overall, the characteristics of myocardial energy metabolism in HCM are complex and multifactorial, involving a shift towards glucose utilization and alterations in mitochondrial function. Further research is needed to fully understand the mechanisms underlying these changes and their impact on HCM pathophysiology and prognosis.

## Strength and limitations

The study is based on a large sample size of 713 patients with HOCM, which enhances the study's statistical power. The study investigates a novel biomarker, the TyG index, and its potential relationship with HOCM prognosis, which may be useful in identifying patients at lower risk of adverse cardiovascular events, and provides a robust assessment of the relationship between the TyG index and HOCM prognosis. The study incorporates myocardial perfusion imaging and metabolic imaging to assess glucose metabolism in the ventricular septum of HOCM patients, which provides insight into the underlying mechanisms.

The study's findings are only applicable to HOCM patients without diabetes, which limits the generalizability of the results to the broader population. The study design is retrospective and observational, which limits its ability to establish causality between the TyG index and HOCM prognosis. The study does not control for potential confounding factors, such as other comorbidities and medications, which may impact the relationship between the TyG index and HOCM prognosis. The study does not investigate the effectiveness of interventions targeting glucose metabolism in improving outcomes for HOCM patients.

## Data Availability

Not applicable.
